# Supplementary material for: Modeling glioblastoma heterogeneity as a dynamic network of cell states
Source: Mol Syst Biol. 2021 Sep 16;17(9):e10105. doi: 10.15252/msb.202010105 (PMC8444284; doi:10.15252/msb.202010105)
Supplement: Supplementary file 6 — Source Data for Figure 5 [file MSB-17-e10105-s004.zip › Figure5A_sourcedata/GSEA_3071/hallmarks_stateIV.GseaPreranked.1623742551702/HALLMARK_KRAS_SIGNALING_DN.html]

Details for gene set HALLMARK\_KRAS\_SIGNALING\_DN[GSEA]

|  || Dataset | state53071 |
| Phenotype | NoPhenotypeAvailable |
| Upregulated in class | na\_pos |
| GeneSet | HALLMARK\_KRAS\_SIGNALING\_DN |
| Enrichment Score (ES) | 0.4633361 |
| Normalized Enrichment Score (NES) | 1.9791468 |
| Nominal p-value | 0.0021691974 |
| FDR q-value | 0.0059719137 |
| FWER p-Value | 0.019 |
Table: GSEA Results Summary

  

Fig 1: Enrichment plot: HALLMARK\_KRAS\_SIGNALING\_DN      
 Profile of the Running ES Score & Positions of GeneSet Members on the Rank Ordered List

  

| PROBE | GENE SYMBOL | GENE\_TITLE | RANK IN GENE LIST | RANK METRIC SCORE | RUNNING ES | CORE ENRICHMENT || 1 | IGFBP2 |  |  | 16 | 2.364 | 0.0894 | Yes |
| 2 | KLHDC8A |  |  | 73 | 1.710 | 0.1457 | Yes |
| 3 | TGFB2 |  |  | 108 | 1.511 | 0.1984 | Yes |
| 4 | KCNQ2 |  |  | 116 | 1.492 | 0.2554 | Yes |
| 5 | TFAP2B |  |  | 134 | 1.433 | 0.3082 | Yes |
| 6 | BTG2 |  |  | 200 | 1.260 | 0.3451 | Yes |
| 7 | PTPRJ |  |  | 320 | 1.006 | 0.3619 | Yes |
| 8 | CELSR2 |  |  | 428 | 0.875 | 0.3757 | Yes |
| 9 | DCC |  |  | 472 | 0.835 | 0.4003 | Yes |
| 10 | SLC25A23 |  |  | 613 | 0.717 | 0.4017 | Yes |
| 11 | CLSTN3 |  |  | 614 | 0.717 | 0.4297 | Yes |
| 12 | CNTFR |  |  | 652 | 0.689 | 0.4497 | Yes |
| 13 | GAMT |  |  | 747 | 0.634 | 0.4566 | Yes |
| 14 | SNN |  |  | 877 | 0.569 | 0.4543 | Yes |
| 15 | CCDC106 |  |  | 943 | 0.546 | 0.4633 | Yes |
| 16 | SKIL |  |  | 1091 | 0.495 | 0.4547 | No |
| 17 | YPEL1 |  |  | 1187 | 0.470 | 0.4551 | No |
| 18 | MTHFR |  |  | 2033 | 0.292 | 0.3058 | No |
| 19 | SHOX2 |  |  | 2186 | 0.269 | 0.2874 | No |
| 20 | PAX3 |  |  | 2233 | 0.264 | 0.2890 | No |
| 21 | ARPP21 |  |  | 2257 | 0.261 | 0.2948 | No |
| 22 | EFHD1 |  |  | 2326 | 0.251 | 0.2917 | No |
| 23 | GTF3C5 |  |  | 2473 | -0.267 | 0.2744 | No |
| 24 | MX1 |  |  | 2779 | -0.303 | 0.2282 | No |
| 25 | PLAG1 |  |  | 2804 | -0.306 | 0.2357 | No |
| 26 | LFNG |  |  | 3991 | -0.509 | 0.0300 | No |
| 27 | CAMK1D |  |  | 4042 | -0.523 | 0.0410 | No |
| 28 | IFI44L |  |  | 4387 | -0.631 | 0.0003 | No |
| 29 | TENM2 |  |  | 4876 | -0.940 | -0.0557 | No |
| 30 | GPRC5C |  |  | 4884 | -0.948 | -0.0200 | No |
| 31 | COPZ2 |  |  | 5071 | -1.227 | -0.0073 | No |
| 32 | EDN1 |  |  | 5081 | -1.246 | 0.0397 | No |
Table: GSEA details [plain text format]

  

Fig 2: HALLMARK\_KRAS\_SIGNALING\_DN: Random ES distribution      
 Gene set null distribution of ES for **HALLMARK\_KRAS\_SIGNALING\_DN**
